# Temporal trends and characteristics of clinical trials for which only one racial or ethnic group is eligible

**DOI:** 10.1016/j.conctc.2018.01.004

**Published:** 2018-01-31

**Authors:** Brian L. Egleston, Omar Pedraza, Yu-Ning Wong, Candace L. Griffin, Eric A. Ross, J. Robert Beck

**Affiliations:** a Fox Chase Cancer Center, Temple University Health System, 333 Cottman Ave., Philadelphia, PA 19111, USA; b Johns Hopkins University, Baltimore, MD, USA

**Keywords:** Clinical trials, Eligibility requirements, Neighborhood characteristics, Protocols

## Abstract

**Background:**

Increasing diversity in clinical trials may be worthwhile. We examined clinical trials that restricted eligibility to a single race or ethnicity.

**Methods:**

We reviewed 19,246 trials registered on ClinicalTrials.gov through January 2013. We mapped trial ZIP-codes to U.S. Census and American Community Survey data. The outcome was whether trials required participants to be from a single racial or ethnic group.

**Results:**

In adjusted analyses, the odds of trials restricting eligibility to a single race/ethnicity increased by 4% per year (95% CI 1.01–1.08, p = .024). Behavioral (5.79% with single race/ethnicity requirements), skin-related (4.49%), and Vitamin D (6.14%) studies had higher rates of single race/ethnicity requirements. Many other trial-specific characteristics, such as funding agency and region of the U.S. in which the trial opened, were associated with eligibility restrictions. In terms of neighborhood characteristics, studies with single race eligibility requirements were more likely to be located in ZIP-codes with greater percentages of those self-reporting the characteristic. For example, 35.2% (SD = 24.9%) of the population self-reported themselves as Black or African American in ZIP-codes with trials requiring participants to be Black/African American, but only 5.9% (SD = 6.9%) self-reported themselves as Black/African American in ZIP-codes with trials that required Asian ethnicity. In ZIP-codes with trials requiring Asian ethnicity, 24.6% (SD = 16.2%) self-reported as Asian. In ZIP-codes with trials requiring Hispanic/Latino ethnicity, 33.3% (SD = 28.5%) self-reported as Hispanic/Latino. Neighborhood level poverty rates and reduced English language ability were also associated with more single race eligibility requirements.

**Conclusions:**

In selected fields, there has been a modest temporal increase in single race/ethnicity inclusion requirements. Some studies may not fall under regulatory purview and hence may be less likely to include diverse samples. Conversely, some eligibility requirements may be related to health disparities research. Future work should examine whether targeted enrollment criteria facilitates development of personalized medicine or reduces trial access.

## 1 Introduction

There has been increasing emphasis on ensuring diversity in clinical trials such that clinical trial results are more generalizable to a broad population [1,2]. However, diverse trials can increase heterogeneity in estimators, which reduces power to detect treatment effects [3,4]. In contrast, less diverse samples reduce variability at the expense of increasing bias with respect to the applicability of study findings to a wider group. This represents a classic bias-variance trade-off [5]. Less diverse samples reduce variability at the expense of increasing bias with respect to the applicability of study findings to a wider group. More diverse samples reduce bias, but at the expense of making studies less likely to achieve their primary endpoints.

Federal agencies have issued policy statements recommending that diverse populations be included in clinical trials [[6], [7], [8]]. Diverse clinical trials not only allow for investigating the generalizability of therapies when applied to a broader population, they allow for planned hypothesis testing for identification of subgroups in which therapies are particularly beneficial [9].

To date, the degree to which diversity is increased in clinical trials has been hampered by incomplete reporting of racial, ethnic, and sex distributions of participants in clinical trials [10]. However, inclusion of diverse samples in clinical trials does seem achievable [[11], [12], [13]], and there is some evidence that diversity in clinical trials is slowly improving [10].

Still, there may be situations where eligibility restrictions may be necessary to meet a study's specific aims. With the rise of “personalized medicine” and genetic screening, there is increased recognition that racial and genetic differences would impact the response to many therapies [9]. In addition, certain behavioral interventions may be adapted to improve health outcomes in certain underserved minority groups [14].

Given potentially conflicting goals of generalizability versus personalized medicine in the setting of an increasingly diverse patient population, understanding patterns of clinical trial eligibility based on race seems important. We used the ClinicalsTrials.gov database to examine studies that require participants to be from one racial or ethnic group, and describe whether there are certain clinical trial characteristics that are associated with these eligibility criteria. As stated on the website, the ClinicalTrials.gov database has over 100,000 registered clinical trials from around the world sponsored by the National Institutes of Health, other public agencies, and private organizations. The National Library of Medicine of the National Institutes of Health (NIH) currently has responsibility for ClinicalTrials.gov. In 2004, members of the International Committee of Medical Journal Editors wrote joint statements requiring clinical trials be prospectively registered for the study results to be considered for publication in their clinical journals [15]. Since that time, the United States Health and Human Services has expanded requirements for public registration of clinical trials [16]. For these reasons, ClinicalTrials.gov likely provides the most comprehensive database of clinical trial inclusion and exclusion requirements [17,18].

As an exploratory study, we examined the relationship of racial exclusions with trial level descriptive fields available in ClinicalTrials.gov such as funders, eligible ages, phase of study, among other characteristics. We also examined neighborhood level characteristics of the centers opening trials, such community racial and ethnic demographics, as well as the poverty rates and English-fluency characteristics of neighborhood residents.

Similar methods were used in a prior report of characteristics of trials that exclude based on English language ability [18]. This study primarily differs from the previous study in that our outcomes consist of whether studies have racial or ethnic eligibility restrictions, rather than English fluency eligibility criteria. Previously, we had found relatively high rates of inclusion criteria stating that participants were required to be fluent in English.

## 2 Methods

By using the ClinicalTrials.gov search algorithm available at the time, we downloaded information from 68,188 clinical trials located in the United States on January 31, 2013, shortly after receiving notification of funding for this work.

We used the sample() permutation command in R (R foundation for Statistical Computing, Vienna Austria) to randomly reorder trials and we examined the inclusion and exclusion criteria of the first 10,361 protocols. After reviewing the first set of trials, we noted which types of studies were more likely to have eligibility exclusions. We next enriched the sample by adding a more targeted group of 10,095 protocols in which we chose trials from categories that seemed more likely to have racial or ethnic eligibility restrictions, which broadly included behavioral, dietary supplement, gastric bypass, gene expression, pharmacodynamics, pharmacokinetics, skin, smoking, and Vitamin D search terms. We identified these areas as potentially having restrictions during prior exploratory and hypothesis generating pilot projects [17,18]. There were few enough gastric bypass surgery, skin, smoking, Vitamin D, and pharmacodynamic trials such that we could include all trials that matched the relevant search terms. For the behavioral, dietary supplement, gene expression, and pharmacokinetic trials, we included a random sample of the matched trials. We also examined 389 protocols that had the term “Caucasian” in the protocol. After eliminating duplicates across the three types of samples, we had 19,246 trials. Of these, 47 did not have eligibility criteria listed, so were removed from the sample. This gave us a final sample of 19,199 trials. Supplemental Figure 1 and Table 1 give more details on the sampling methods for trial inclusion.

Our protocol for the study initially called for both the random and enriched targeted sampling strategies. The rationale for including a random sample of trials was that the random sample would allow us to estimate an unbiased proportion with racial eligibility requirements. The rationale for including an enriched targeted sample was that we expected racial exclusions to be a small percentage of the total sample, and we would have more power to investigate associations with the targeted sample. While the prevalence of exclusions would be biased in our targeted sample, the relationships among variables and eligibility requirements (e.g. slopes from regressions) would be unbiased (as per Prentice and Pyke [19]).

This work was funded by a grant from the National Cancer Institute with the aim of examining racial and English fluency exclusions in clinical trials. The preliminary data used to design the study suggested that as few as 1% of studies might have racial restrictions; this was conservative with respect to English language restrictions as we subsequently discovered that rates were substantially greater than 1% [18]. Given that we estimated that racial exclusions might be low, we chose to examine a random sample (i.e. non-targeted based on trial criteria) of at least 10,000 studies such that we would have 90% power to detect odds ratios of 2.0 when comparing trial characteristics with (expected number = 100) and without exclusions (expected number = 9900). We assumed a 5% Type I error rate (2-sided) with a 25% rate of a clinical trial characteristics, such as the U.S. census defined region of the country (i.e. Northeast, Midwest, South, West, Multi-region), in studies that do not have exclusions. In other words, if 25% of trials without exclusions were opened in the Northeastern region of the United States, we would be able to detect an association of racial eligibility criteria with region if 40% of trials with exclusions were located in the Northeast region (40%/60%)/(25%/75%) = OR of 2.0). Hypothesis testing in the second set of enriched targeted trials was considered independent, with similar power.

We defined that a study required participants to be a member of a single race or ethnic group if the eligibility criteria in the inclusion and exclusion fields of ClinicalTrials.gov specified as such. Examples of specific inclusion criteria were requirements that participants be “Caucasian”, “European Descent,” or “African American.” Three individuals coded the studies as described previously [18].

We used generalized Fisher's exact tests and t-tests to examine the relationship of studies requiring participants to be from a single race or ethnic group with trial characteristics for trials open in any year. Due to the sparseness of some of the cells, we felt that Fisher's Exact test would be more reliable; in cases in which the table or sample size was too large to calculate Fisher's Exact test, we instead used Chi-squared tests. ClinicalTrials.gov has fields detailing a trial's funding agency, study type (intervention versus observation), U.S. census defined region of the country, intervention type (e.g. device, drug, or genetic focus, among other types), phase (e.g. I, II, III), age group (children, adults, or all ages), and included genders. We excluded missing data when performing hypotheses tests, although we report the amount of missing data in tables.

We also examined the area level characteristics of clinical trials using ZIP-code level data of institutions either opening or sponsoring trials for those trials opened in 1995 or later. The Zoning Improvement Plan (ZIP) Code is a 5 digit system, with additional 4 digit subdivisions, used by the United States Post Office to geographically partition the United States for ease of mail delivery [20]. We matched the 5-digit ZIP-codes of institutions listed on ClinicalTrials.gov with ZIP-code level demographic characteristics reported by the United States Census or the American Community Survey (ACS) [21]. Area level demographics of interest included the proportion of Census or ACS ZIP-code respondents self-identifying as African American/Black, Caucasian/White, Hispanic, or Asian. Similarly, we examined the proportion within ZIP-codes responding that they had an income below the poverty line, spoke English alone, or spoke English less than very well.

Until 2010, the United States Census asked English language ability and income questions on the census long form distributed to a subset of census respondents; the majority of respondents received a shorter form which included racial and ethnic questions alone. However, the Census long form was discontinued in 2010, and ZIP-code level English language ability and income information was instead captured by the American Community Survey. Hence, we used year 2000 Census ZIP-code level data for studies that opened from 1995 to 2004. We used 2010 Census ZIP-code level data for racial and ethnic information for studies that opened after 2005; we used ACS ZIP-code level data for English language and poverty information for studies that opened after 2005. For the relatively small proportion of studies with missing data in the relevant time period, we used data from the other time period. Many multi-center trials opened in more than one ZIP-code; for these we took a simple average of the multiple ZIP-code level demographics values. In analyses, we excluded the small proportion (approximately 1%) of trials that opened prior to 1995 to reduce estimator variability that could result from matching small numbers of trials to the relevant pre-2000 census data. We used means, medians, and inter-quartile ranges to characterize the ZIP-code level percentage data. Kruskal-Wallis tests were used to examine whether racial eligibility criteria varied among ZIP-code levels with different demographic characteristics.

We were also interested in the potentially non-linear relationships of year and ZIP-code level characteristics with the likelihood of a trial having single race-specific eligibility criteria. To examine such relationships, we used logistic regression models in which we entered year or ZIP-code level percentages using either linear (i.e. untransformed) variables or restricted cubic spline [22] terms with three knots. We used the restricted cubic splines to create figures that showed the general relationship of year and ZIP-code level demographics with the probability of having single race eligibility requirements. Restricted cubic splines are useful because they do not impose strict linearity on the relationship of variables with the log odds of a study having race-specific eligibility criteria.

We used a multiple logistic regression to investigate the relationship of non-ZIP-code trial characteristics with a study having a single race or ethnicity inclusion requirement in the post-1994 internet registration era. Small numbers of specific types of inclusion criteria prevented us from examining multiple regression models more widely. We were able to combine data from the random and targeted samples for the multiple logistic regression since the odds ratios for the regression slope parameters are identified and unbiased for such a design [19,23]. A p-value of less than 5% was used as the criteria for statistical significance.

We used STATA (Statacorp, College Station, TX) for analyses. Since the study did not examine human subjects data but instead simply examined details of trial protocols, the Fox Chase Cancer Center Institutional Review Board determined that the study was not human subjects research. The National Cancer Institute of the United States National Institutes of Health funded this research (grants R03CA167264 and P30CA006927), but the results presented here were not reviewed by the funders. Our dataset is available upon request to the corresponding author.

## 3 Results

### 3.1 Sampling scheme

In Supplemental Figure 1, we present a diagram that describes the numbers of trials identified, removed due to duplication or missing data, and finally examined. We further present in Supplemental Table 1 the distribution of single race/ethnicity inclusion requirements in our random and targeted samples. We compare the targeted inclusion rates with the random sample rate after removing duplicates from the random sample (i.e. those found during both the random and targeted searches). We found that the proportion of studies with single race/ethnicity eligibility requirements differed significantly (p < .05) between the targeted search-word groups and the random sample, with the exception of gastric bypass surgery (p = .27).

### 3.2 Relationships of clinical trial specific characteristics with eligibility

In Table 1, we present characteristics of all the trials combined (targeted and random samples). There is not a clear indication that rates of single race/ethnicity inclusion eligibility have changed over time. Trials that were fully funded by industry, the NIH, or the United States Federal Government had lower rates of eligibility restrictions than those funded by other sources. In terms of intervention type, behavioral studies had by far the most single race/ethnicity eligibility restrictions (5.79%), with 2.97% of behavioral trials requiring participants to be Black or African American. Studies that did not fall within the typical Phase 0–4 paradigm were more likely to have eligibility restrictions. With respect to gender, studies for women had the most eligibility restrictions, with 1.06% of those requiring that participants be Caucasian/White, 1.86% requiring that participants be Black/African American, and 1.01% requiring that participants be Hispanic. In terms of the region of the United States in which the trial is open, 2.86% of the studies in the West had single race/ethnicity eligibility, largely because many trials in the West require participants to be either Asian (1.29%) or Hispanic (0.90%). Trials opening in the South also had relatively higher rates of eligibility restrictions (2.37%), which seems mostly to be driven by a higher proportion of studies that require participants to be Black/African American in the South. We reproduce Table 1 but only for the random sample in Supplemental Table 2.

**Table 1:** Characteristics of trials by presence of racial inclusion type. The p-value compares those trials open to more than one race with those open to just one race. Due to small sample sizes in some cells, it was not necessarily meaningful to compare the trials with different types of exclusions to each other. We excluded missing data from calculations of p-values.

	Open to more than 1 race n (row %)	White Only n (row %)	Black Only n (row %)	Asian Only n (row %)	Hispanic Only n (row %)	P-value
**Number**	18,859	66	147	57	70	
**Year Opened**						.083
Before 1995	148 (98.67)	2 (1.33)	0 (0)	0 (0)	0 (0)	
1995–1999	728 (99.05)	1 (0.14)	4 (0.54)	0 (0)	2 (0.27)	
2000–2004	3028 (98.57)	4 (0.13)	27 (0.88)	7 (0.23)	6 (0.20)	
2005–2009	8364 (98.18)	26 (0.31)	64 (0.75)	27 (0.32)	38 (0.45)	
2010 and later	6131 (97.92)	33 (0.53)	51 (0.81)	22 (0.35)	24 (0.38)	
Missing	460 (99.57)	0 (0)	1 (0.22)	1 (0.22)	0 (0)	
**Funding Agency**						<.001
Industry	5297 (98.97)	21 (0.39)	5 (0.09)	28 (0.52)	1 (0.02)	
NIH	2025 (98.49)	7 (0.34)	15 (0.73)	1 (0.05)	8 (0.39)	
U.S. Federal Government	347 (98.86)	0 (0)	2 (0.57)	0 (0)	2 (0.57)	
Combination of the above	5739 (97.89)	17 (0.29)	62 (1.06)	13 (0.22)	32 (0.55)	
Other	5451 (97.74)	21 (0.38)	63 (1.13)	15 (0.27)	27 (0.48)	
**Study Type**						.51
Expanded Access	16 (100)	0 (0)	0 (0)	0 (0)	0 (0)	
Interventional	16,087 (98.27)	48 (0.29)	122 (0.75)	50 (0.31)	64 (0.39)	
Observational	2756 (98.01)	18 (0.64)	25 (0.89)	7 (0.25)	6 (0.21)	
**Intervention**						<.001
Behavioral	2536 (94.21)	3 (0.11)	80 (2.97)	22 (0.82)	51 (1.89)	
Biological	1133 (99.91)	0 (0)	0 (0)	1 (0.09)	0 (0)	
Device	771 (99.61)	3 (0.39)	0 (0)	0 (0)	0 (0)	
Dietary Supplement	1060 (97.61)	8 (0.74)	16 (1.47)	1 (0.09)	1 (0.09)	
Drug	9645 (99.06)	29 (0.30)	25 (0.26)	28 (0.29)	10 (0.10)	
Genetic	143 (98.62)	1 (0.69)	0 (0)	1 (0.69)	0 (0)	
Other	2740 (98.10)	18 (0.64)	24 (0.86)	4 (0.14)	7 (0.25)	
Procedure	739 (99.06)	4 (0.54)	2 (0.27)	0 (0)	1 (0.13)	
Radiation	92 (100)	0 (0)	0 (0)	0 (0)	0 (0)	
**Phase**						<.001
Phase 0	157 (97.52)	1 (0.62)	3 (1.86)	0 (0)	0 (0)	
Phase 1	3561 (98.59)	15 (0.42)	10 (0.28)	24 (0.66)	2 (0.06)	
Phase 1 | Phase 2	982 (99.09)	1 (0.10)	4 (0.40)	2 (0.20)	2 (0.02)	
Phase 2	3995 (99.45)	5 (0.12)	9 (0.22)	2 (0.05)	6 (0.15)	
Phase 2 | Phase 3	325 (97.89)	2 (0.60)	0 (0)	2 (0.60)	3 (0.90)	
Phase 3	1892 (98.85)	3 (0.16)	14 (0.73)	4 (0.21)	1 (0.05)	
Phase 4	1211 (97.66)	5 (0.40)	17 (1.37)	0 (0)	7 (0.56)	
Other	6736 (97.17)	34 (0.49)	90 (1.3)	23 (0.33)	49 (0.71)	
**Age groups**						.071
Adults or Seniors	14,944 (98.21)	59 (0.39)	108 (0.71)	50 (0.33)	56 (0.37)	
Children only	2834 (98.61)	4 (0.14)	26 (0.90)	2 (0.07)	8 (0.28)	
All ages	1081 (97.56)	3 (0.27)	13 (1.17)	5 (0.45)	6 (0.54)	
**Gender**						<.001
Both	16,216 (98.6)	38 (0.23)	101 (0.61)	46 (0.28)	46 (0.28)	
Female	1809 (95.97)	20 (1.06)	35 (1.86)	2 (0.11)	19 (1.01)	
Male	834 (96.19)	8 (0.92)	11 (1.27)	9 (1.04)	5 (0.58)	
**Region Opened**						<.001
Midwest	2755 (98.15)	16 (0.57)	29 (1.03)	4 (0.14)	3 (0.11)	
Northeast	3665 (98.18)	15 (0.40)	35 (0.94)	4 (0.11)	14 (0.38)	
South	4703 (97.63)	12 (0.25)	65 (1.35)	8 (0.17)	29 (0.60)	
West	2476 (97.14)	9 (0.35)	8 (0.31)	33 (1.29)	23 (0.90)	
Multi-region	4972 (99.52)	9 (0.18)	10 (0.20)	4 (0.08)	1 (0.02)	
Missing	288 (96.97)	5 (1.68)	0 (0)	4 (1.35)	0 (0)	

**Fig. 1:**
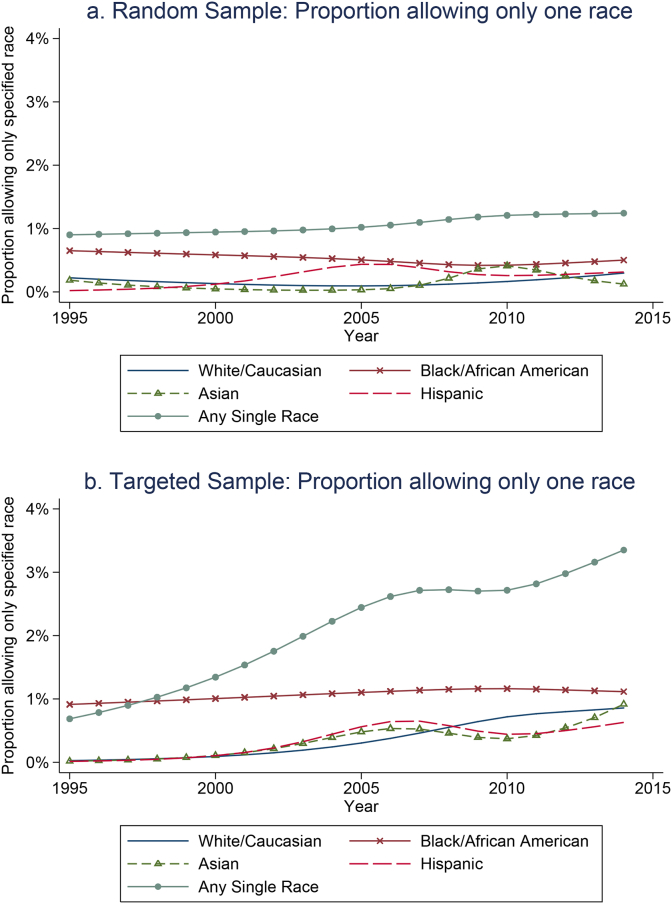
a and b: Change in race-specific inclusion rates over time. Notes: Random sample p-values for testing whether rates differ by year using logistic regressions with linear terms: p = .503 for White/Caucasian, p = .467 for Black/African American, p = .018 for Asian, p = .741 for Hispanic ethnicity, p = .344 for any single race/ethnicity inclusion requirement. Enriched targeted sample: p = .002 for White/Caucasian, p = .695 for Black/African American, p = .246 for Asian, p = .339 for Hispanic ethnicity, p = .010 for any single race/ethnicity inclusion requirement.

In Fig. 1, we present temporal trends for the relationship of studies that require participants to be from a single race or ethnicity. In the random sample (Fig. 1a), there is limited evidence that rates of eligibility requirements have changed since 1995. However, in the targeted sample (Fig. 1b), the number of trials with single race eligibility requirements has increased from 1% in 1995 to approximately 3% in 2013 (p = .010). In terms of race-specific trends driving this overall growth in studies with eligibility requirements, the relationship of year with trials that require participants to be White/Caucasian was the only statistically significant trend.

### 3.3 Relationships of neighborhood level characteristics with eligibility

In Table 2, we present the ZIP-code level characteristics associated with single race and ethnicity inclusion requirements, studies with single race/ethnicity requirements were more likely to be located in ZIP-codes with greater percentages of those self-reporting the characteristic. For example, an average of 57.7% (Standard Deviation [SD] = 18.2%) of the population self-reported themselves to be White or Caucasian alone in areas with trials that required participants to be White or Caucasian. However, only an average of 41.4% (SD = 23.9%) of the population self-reported that they were White or Caucasian in areas with trials requiring participants to be Black or African American. Similarly, an average of 35.2% (SD = 24.9%) of the population self-reported themselves as Black or African American in ZIP-codes with trials requiring participants to be Black/African American, but only 5.9% (SD = 6.9%) of the population self-reported themselves to be Black/African American in ZIP-codes with trials that require participants to be Asian.

**Table 2:** Demographic characteristics of the ZIP-codes by racial inclusion type. Trials with missing ZIP-code data were excluded. SD = standard deviation, IQR = inter-quartile range, n = Number of trials with non-missing data. The statistics summarize the percents among ZIP-codes.

	Open to more than 1 race	White Only	Black Only	Asian Only	Hispanic Only	Kruskal-Wallis P-value
Percent responding that they are White alone in ZIP-code	n = 14,593	n = 53	n = 123	n = 36	n = 54	0.0001
Mean (SD)	56.2% (20.8%)	57.7% (18.2%)	41.4% (23.9%)	52.4% (18.4%)	43.9% (25.8%)	
Median (IQR)	58.8% (43.5%,71.0%)	64.7% (42%,71.8%)	36.6% (19.5%,61%)	58.4% (40.4%,62.7%)	56.1% (18.3%,64.3%)	
Percent responding that they are Black/African American alone in ZIP-code	n = 14,593	n = 53	n = 123	n = 36	n = 54	0.0001
Mean (SD)	17.4% (17.8%)	18.7% (18.2%)	35.2% (24.9%)	5.9% (6.9%)	10.6% (12.5%)	
Median (IQR)	11.5% (5%,23%)	12.8% (5.6%,25.7%)	32.7% (14.8%,50.7%)	3.8% (1.5%,6.1%)	6.1% (3.5%,11.3%)	
Percent responding that they are Asian alone in ZIP-code	n = 14,593	n = 53	n = 123	n = 36	n = 54	0.0001
Mean (SD)	9.7% (9.4%)	10.5% (8.6%)	8.9% (8%)	24.6% (16.2%)	9.5% (8.1%)	
Median (IQR)	7.3% (3.9%,12.7%)	9.7% (3.5%,14.8%)	6.0% (2.7%,14.8%)	18.3% (15%,31.1%)	7.0% (2.8%,16%)	
Percent responding that they are Hispanic alone in ZIP-code	n = 14,593	n = 53	n = 123	n = 36	n = 54	0.0001
Mean (SD)	13.7% (15%)	10% (12.7%)	12% (17.1%)	11.6% (9.7%)	33.3% (28.5%)	
Median (IQR)	9% (4.6%,15.8%)	4.2% (3.2%,10.3%)	4.7% (2.8%,13.9%)	10.1% (5.2%,14.6%)	18.9% (10.2%,53.7%)	
Percent responding that they speak English only in ZIP-code	n = 14,526	n = 53	n = 123	n = 35	n = 54	0.0001
Mean (SD)	75.7% (14.6%)	78.8% (13.7%)	76.2% (18.2%)	60.2% (18.9%)	57.8% (22.1%)	
Median (IQR)	78.3% (69.9%,85.9%)	80.8% (75.3%,86.2%)	80.9% (69.2%,89.5%)	63.7% (43.5%,72.9%)	68.2% (42.7%,70.2%)	
Percent responding that they speak English less than “very well” in ZIP-code	n = 14,526	n = 53	n = 123	n = 35	n = 54	0.0001
Mean (SD)	8.8% (8.2%)	7.3% (7.9%)	10% (11.7%)	19.2% (11.3%)	17.6% (14.1%)	
Median (IQR)	4.5% (2.4%,9.2%)	5.7% (2.3%,12.6%)	17.3% (11%,32.9%)	14% (6.6%,23.8%)	0% (0%,0%)	
Poverty Rate in ZIP-code	n = 14,120	n = 37	n = 101	n = 34	n = 52	0.0001
Mean (SD)	22.4% (15.2%)	29.7% (19.6%)	28.2% (17.4%)	17.5% (16.7%)	21.2% (12.4%)	
Median (IQR)	18.9% (11.8%,29.8%)	28.6% (13.3%,39.7%)	27.2% (14.6%,36.9%)	12% (10.8%,21.0%)	19.4% (10.5%,31%)	

Also in Table 2, we examine the relationship of single race/ethnicity inclusion requirements with self-reported English language ability and poverty rates. The average percentage of respondents that speak English only is lower in ZIP-codes with trials that require participants to be Asian or Hispanic. For example, 60.2% (SD = 18.9%) of respondents reported speaking English only in areas with Asian only trials, compared with 78.8% (SD = 13.7%) in areas with White/Caucasian only trials and 76.2% (SD = 18.2%) in areas with Black/African American trials. A similar trend is seen with those who speak English less than very well. For trials that require participants to be Hispanic, 17.6% (SD = 14.1%) of ZIP-code residents report speaking English less than very well, versus 8.8% (SD = 8.2%) for trials open to two or more races/ethnicities. In terms of poverty, ZIP-codes with trials that required participants to be Asian had the lowest poverty rates on average (17.5%, SD = 16.7%) compared with 28.2% (SD = 17.4%) with trials that required participants to be Black/African American.

In Supplemental Table 3, we repeat the analyses presented in Table 2, but only include the random sample of participants.

**Fig. 2:**
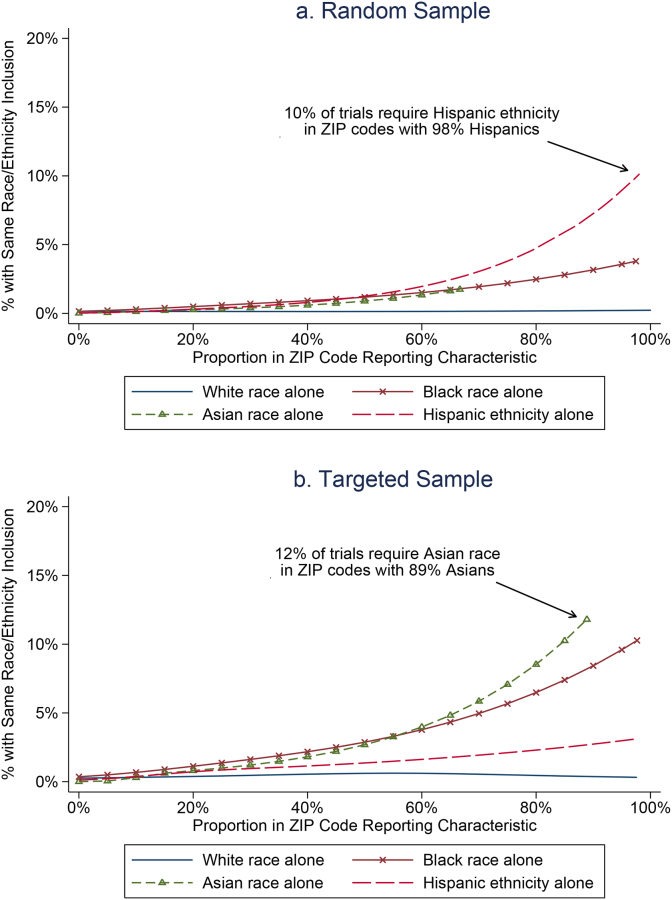
a and b: Relationship of ZIP-code level racial demographics with the percentage of clinical trials in ZIP-codes having specified inclusion restrictions. Here, we match the proportion of the population self-reporting a race/ethnicity with the proportion of trials having the same race/ethnicity requirement. Examples of interpretation are given in the figures. Notes: Random sample p-values for testing whether slopes differ using logistic regressions with linear terms: p < .001 for Hispanic relationship, p < .001 for Asian relationship, p < .001 for Black relationship, p = .773 for White relationship. Enriched targeted sample: p < .001 for Hispanic relationship, p < .001 for Asian relationship, p < .001 for Black relationship, p = .752 for White relationship.

In Fig. 2, we more closely examine the relationship of ZIP-code level race and ethnicity demographics with eligibility requirements. Here we plot the proportion of residents in each ZIP-code self-reporting a specific race or ethnicity (x-axis) and the estimated percent of trials in the ZIP-code requiring participants to be the same race or ethnicity. In Fig. 2a, we present the relationships for the random sample of trials. The top dashed line indicates that as the proportion of residents in a ZIP-code that are Hispanic increases (x-axis), the proportion of trials that require participants to be Hispanic also increases (p < .001 for test of slope from a logistic regression). We see similar statistically significant relationships between the proportion of residents who are Asian and the proportion of studies requiring participants to be Asian, and the proportion of residents who are Black/African American and the proportion of trials requiring participants to be Black/African American. The relationship of the proportion of residents who are White/Caucasian is not related to the proportion of studies in a ZIP-code that require participants to be White/Caucasian. In Fig. 2b, we present the corresponding results for the targeted sample; the inferences are very similar.

**Fig. 3:**
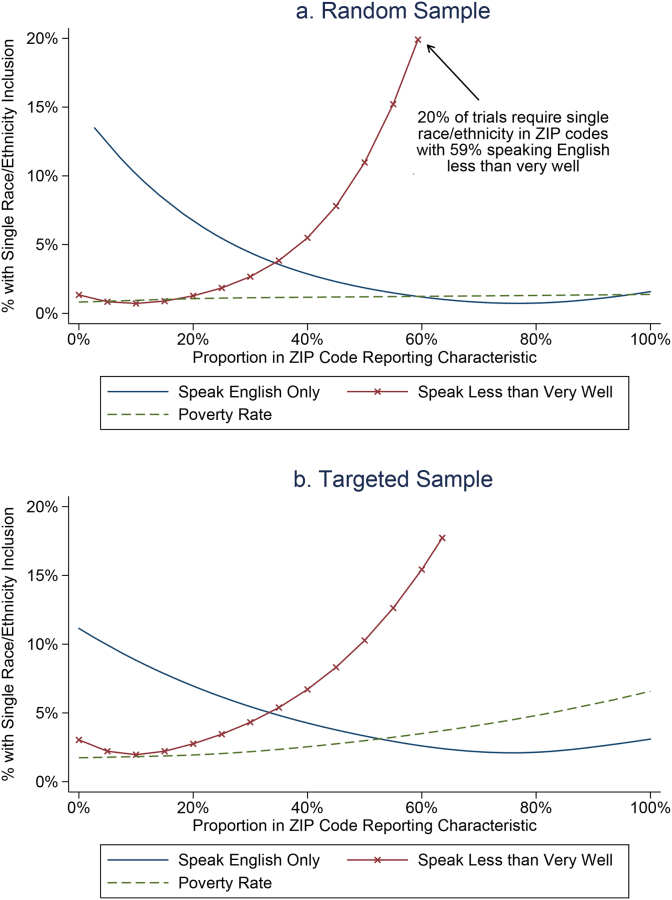
a and b: Relationship of ZIP-code level English-fluency characteristics or poverty rates with the percentage of clinical trials in ZIP-codes that have any single race/ethnicity inclusion requirement. We give an example of interpretation in Figure 3a. Notes: Random sample p-values for testing whether slopes differ using logistic regressions with linear terms: p < .001 for speak English Only, p < .001 speak less than very well, and p = .405 for poverty rate associations. Enriched targeted sample: p = .001 for speak English Only, p < .001 speak less than very well, and p = .002 for poverty rate associations.

In Fig. 3, we examine the relationship of eligibility requirements with ZIP-code English language fluency and poverty rate. In the random sample data of Fig. 3a, we graph the proportion of trials that have any single race or ethnicity inclusion requirements by the percentage in a ZIP code who speaks English only, by the percentage who speaks English less than very well, and by the poverty rate. There seems to be a strong relationship between English language ability and the probability of a trial having an eligibility requirement. In the random sample, ZIP-code level poverty rates were not associated with racial or ethnic inclusion requirements. Fig. 3b demonstrates that the relationship between poverty rates and clinical trial inclusion criteria became more salient and statistically significant in the targeted sample.

In Supplemental Table 4, we present the multiple logistic regression results for the combined sample after 1995. In the regression, year was statistically significant after controlling for the other characteristics, with the odds of a study restricting eligibility to a single race increasing by 4% per year (95% odds ratio CI 1.01–1.08, p = .024). Also in the model, industry sponsors seemed to have higher rates of eligibility restrictions than other studies. Behavioral studies by far had the greatest odds of having eligibility restrictions. The overlapping confidence intervals suggest that the phase of the trial was not highly related to the odds of having eligibility restrictions. Studies with a target gender (as compared to those who included both genders) had much greater odds of having eligibility restrictions. Finally, studies open in multiple regions were not as likely to have eligibility restrictions as those that open in a single region. The year effect was equivalent when refitting the model but including pre-1995 studies (odds ratio = 1.04, p = .028).

## 4 Discussion

We found evidence that the proportion of studies with eligibility requirements that participants be a single race or ethnicity has increased slightly over time, with such increase driven by a group of studies with certain focus areas, such as behavioral interventions, skin related studies, Vitamin D research, smoking related trials, and dietary supplements. Studies that were funded by government agencies were generally less likely to have single race eligibility requirements. This could be due to government funded studies being more likely to fall under federal regulations, such as those developed by the Food and Drug Administration [7] or NIH [8], that encourage greater diversity in trials. We found that rates of racial eligibility requirements were substantially lower than rates of English fluency requirements, as reported earlier [18].

In adjusted analysis, behavioral intervention trials had by far the highest rates of eligibility restrictions, while industry funded trials also had relatively high rates of inclusion requirements. Studies that targeted a single gender had higher adjusted rates of eligibility restrictions. Studies that fell outside of the Phase 1–4 paradigm were more likely to have racial eligibility requirements in unadjusted, but not adjusted, analyses. However, studies that were open in multiple regions of the United States had much lower odds of having racial eligibility restrictions than studies opened in a single region. Interestingly, industry sponsored trials were more likely to have racial eligibility restrictions only after controlling for other trial characteristics; this could indicate that some industry trials are related to the investigation of how interventions may affect racial subgroups (e.g. ClinicalTrials.gov ID NCT01523392, NCT00551317).

Some of these findings could be due to contradictory factors influencing eligibility restrictions. It is possible that some studies do not fall under regulatory purview and are hence less likely to include a diverse sample as per regulatory requirements [[6], [7], [8]]. Conversely, the relatively high number of behavioral trials that restrict eligibility to participants of Black/African American race or Hispanic ethnicity may be due to behavioral interventions designed to reduce health disparities among these populations. ZIP-code findings may suggest that racial eligibility requirements may be due to efforts to reduce health disparities, as the percentage of residents meeting racial requirements was larger in ZIP-codes with such requirements.

Some studies on ClinicalTrials.gov do give reasons for restricting eligibility. A stated reason for restricting trials to those of European ancestry, for example, include reducing “ethnic admixture that could bias the genetic analysis” (ClincialTrials.gov ID NCT01202955). Reasons for including some ethnic groups are often related to addressing health problems which disproportionately affect such groups, such as Type 2 diabetes in African Americans (e.g. ClinicalTrials.gov ID NCT01324011). Behavioral studies sometimes limit enrollment to certain groups in order to develop culturally-relevant interventions, such as smoking cessation programs tailored for self-identified ethnic Chinese (ClinicalTrials.gov ID NCT00714467), or diabetes self-management programs for Mexican-Americans (ClinicalTrials.gov ID NCT01238289). However, many studies do not give the reason for limiting eligibility to a single group (e.g. ClinicalTrials.gov ID NCT00698984, NCT00990873 prior to 2016, NCT01465360).

There has been increasing research documenting the degree to which health disparities are affecting outcomes in the United States [24,25]. Concurrently, there has been an increased focus on “dissemination and implementation” research with a goal of spreading evidence-based practices and interventions into policy designs and community settings [26]. A focus on interventions that serve a single racial or ethnic group may be at odds with implementation and dissemination goals that seek to generalize interventions so that they are applicable to broad populations. Further, restricting eligibility to certain racial or ethnic groups might underestimate the heterogeneity of individuals or cultures within a single group [27]. In addition to the goals of reducing disparities and increasing dissemination, there is interest in using racial and ethnic genetic differences to help provide individually personalized medicine [9]. Our work did not focus on predicting whether more personalized and restricted eligibility requirements or more open and inclusive, and hence more disseminable, eligibility requirements will be seen in the future. However, we do provide insight into the degree to which certain types of restrictive eligibility requirements may be increasing.

Limitations include the fact that our race and ethnicity definitions varied across trials. For example, some studies included “Latinos” and “Hispanics (e.g. NCT01457066) while others included people with heritage from only certain countries, such as Mexico (e.g. NCT01168765). If the percentage of studies with single race/ethnicity eligibility requirements is indeed increasing, as our data suggest, than future studies may investigate if relationships vary after defining more specific race and ethnicity subgroups. Another limitation is the possibility that eligibility criteria on ClinicalTrials.gov differed from the IRB approved protocols actually used. While we restricted our search to studies using the region locator on ClinicalTrials.gov, some studies may have never opened enrollment at locations within the United States. Further, some institutional ZIP-codes were business ZIP-codes which did not contain any demographic information on the surrounding community. Despite these limitations, our study was strengthened by our rigor selecting both random and enriched targeted samples.

In summary, we found that the proportion of studies requiring participants to be a single race or ethnicity has been modestly increasing over time in a subset of scientific areas. The stated reasons for such restrictions are diverse. Future work can examine whether targeted enrollment criteria facilitates development of personalized medicine or reduces access to trials.

## 5 Compliance with ethical standards

The authors report no conflicts of interest. Since the study did not examine human subjects data but instead simply examined details of trial protocols along with aggregated Census data, the Fox Chase Cancer Center Institutional Review Board determined that the study was not human subjects research. Informed consent for humans was hence not required as there was no data obtained at the individual human subject level.

## Appendix A Supplementary data

mmc1 mmc1

mmc2 mmc2

## References

[1] Elting L.S., Cooksley C., Bekele B.N., Frumovitz M., Avritscher E.B., Sun C., Bodurka D.C., Generalizability of cancer clinical trial results: prognostic differences between participants and nonparticipants (2006). Cancer.

[2] Tunis S.R., Stryer D.B., Clancy C.M. (2003). Practical clinical trials: increasing the value of clinical research for decision making in clinical and health policy. J. Am. Med. Assoc..

[3] Rosenbaum P.R. (2005). Heterogeneity and causality: unit heterogeneity and design sensitivity in observational studies. Am Stat.

[4] Maitournam A., Simon R. (2005). On the efficiency of targeted clinical trials. Stat. Med..

[5] Briscoe E., Feldman J. (2011). Conceptual complexity and the bias/variance tradeoff. Cognition.

[6] (February 27, 2003). AHRQ Policy on the Inclusion of Priority Populations in Research.

[7] United States Government Accountability Office (September 28 2007). Prescription Drugs: FDA Guidance and Regulations Related to Data on Elderly Persons in Clinical Drug Trials.

[8] NIH Policy and Guidelines on The Inclusion of Women and Minorities as Subjects in Clinical Research – Amended, October, 2001. As accessed on August 10, 2017, at: http://grants.nih.gov/grants/funding/women_min/guidelines_amended_10_2001.htm.

[9] Ortega V.E., Meyers D.A. (2014). Pharmacogenetics: implications of race and ethnicity on defining genetic profiles for personalized medicine. J. Allergy Clin. Immunol..

[10] Kwiatkowski K., Coe K., Bailar J.C., Swanson G.M. (2013). Inclusion of minorities and women in cancer clinical trials, a decade later: have we improved?. Cancer.

[11] Adolescent type 1 Diabetes cardio-renal Intervention Trial Research Group (2009). Adolescent type 1 diabetes cardio-renal intervention trial (AdDIT). BMC Pediatr..

[12] Inman M., Daneman D., Curtis J., Sochett E., Elia Y., Dunger D.B., Deanfield J., Mahmud F.H. (2016). Assessing social determinants of health in a pediatric diabetes research trial: Are recruited subjects representative of the larger clinical population?. Diabetes Res. Clin. Pract..

[13] Langford A.T., Resnicow K., Dimond E.P., Denicoff A.M., St Germain D., McCaskill-Stevens W., Enos R.A., Carrigan A., Wilkinson K., Go R.S. (2014). Racial/ethnic differences in clinical trial enrollment, refusal rates, ineligibility, and reasons for decline among patients at sites in the National Cancer Institute's Community Cancer Centers Program. Cancer.

[14] Nápoles A.M., Santoyo-Olsson J., Stewart A.L. (2013). Methods for translating evidence-based behavioral interventions for health-disparity communities. Prev. Chronic Dis..

[15] De Angelis C.D., Drazen J.M., Frizelle F.A., Haug C., Hoey J., Horton R., International Committee of Medical Journal Editors (2005). Is this clinical trial fully registered?--A statement from the International Committee of Medical Journal Editors. N. Engl. J. Med..

[16] https://www.federalregister.gov/documents/2016/09/21/2016-22129/clinical-trials-registration-and-results-information-submission as accessed on August 10, 2017.

[17] Egleston B.L., Dunbrack R.L., Hall M.J. (2010). Clinical trials that explicitly exclude gay and lesbian patients. N. Engl. J. Med..

[18] Egleston B.L., Pedraza O., Wong Y.N., Dunbrack R.L., Griffin C.L., Ross E.A., Beck J.R. (2015 Dec). Characteristics of clinical trials that require participants to be fluent in English. Clin. Trials.

[19] Prentice R.L., Pyke R. (1979). Logistic disease incidence models and case-control studies. Biometrika.

[20] The United States Postal Service (November 2012). An American History. (Washington D.C.: Government Relations.

[21] https://factfinder.census.gov/faces/nav/jsf/pages/index.xhtml. as accessed on November 3, 2017.

[22] Harrell F.E. (2001). Regression Modeling Strategies.

[23] Hosmer D.W., Lemeshow S. (2000). Applied Logistic Regression.

[24] Adler N.E., Newman K. (2002). Socioeconomic disparities in health: pathways and policies. Health Aff..

[25] Schuster M.A., Elliott M.N., Kanouse D.E., Wallander J.L., Tortolero S.R., Ratner J.A., Klein D.J., Cuccaro P.M., Davies S.L., Banspach S.W. (2012). Racial and ethnic health disparities among fifth-graders in three cities. N. Engl. J. Med..

[26] Purtle J., Peters R., Brownson R.C. (2016). A review of policy dissemination and implementation research funded by the National Institutes of Health, 2007–2014. Implement. Sci..

[27] Weinick R.M., Jacobs E.A., Stone L.C., Ortega A.N., Burstin H. (2004). Hispanic healthcare disparities: challenging the myth of a monolithic Hispanic population. Medical Care.

