# Supplementary material for: Temporal trends and characteristics of clinical trials for which only one racial or ethnic group is eligible
Source: Contemp Clin Trials Commun. 2018 Jan 31;9:135–42. doi: 10.1016/j.conctc.2018.01.004 (PMC5898501; doi:10.1016/j.conctc.2018.01.004)
Supplement: mmc2 [file mmc2.docx]

Supplemental Table 1. Comparison of targeted versus random sample of trials. In the heading parentheses, we list additional more specific search terms used. We use "+" to denote that we searched for the stated term plus the additional term (e.g. "Gastric Bypass Surgery"). The p-values represent the comparison with the non-overlapping random sample for each search word. *The enriched targeted studies are further characterized by search word used to identify them.

| **Sample** | **Open to more than 1 race**  **n (row %)** | **White Only**  **n (row %)** | **Black Only**  **n (row %)** | **Asian Only**  **n (row %)** | **Hispanic Only**  **n (row %)** | **Fisher's P-value** |
| --- | --- | --- | --- | --- | --- | --- |
| **All Studies, removing duplicates** | 18,859 (98.23) | 66 (0.34) | 147 (0.77) | 57 (0.30) | 70 (0.36) |  |
| **By How Study Sampled** |  |  |  |  |  |  |
| Random Sample (with duplicates) | 10,249 (98.92) | 14 (0.14) | 48 (0.46) | 20 (0.19) | 30 (0.29) | NA |
| Targeted non-Caucasian Search Words* | 9,870 (97.77) | 35 (0.35) | 101 (1.00) | 42 (0.42) | 47 (0.47) | <0.001 |
| Targeted Caucasian Search Word*  (+European Ancestry or Descent) | 313 (80.46) | 49 (12.60) | 22 (5.66) | 3 (0.77) | 2 (0.51) | <0.001 |
| Random (removing targeted duplicates) | 8,763 (99.23) | 0 (0) | 28 (0.32) | 14 (0.16) | 21 (0.24) | NA |
| **By Search Word for Targeted Studies** |  |  |  |  |  |  |
| Behavioral | 1,545 (93.64) | 1 (0.06) | 55 (3.33) | 14 (0.85) | 35 (2.12) | <0.001 |
| Dietary Supplement | 1,964 (98.25) | 11 (0.55) | 17 (0.85) | 5 (0.25) | 2 (0.1) | <0.001 |
| Gastric Bypass (+Surgery, +Banding,  Roux en y) | 323 (99.08) | 2 (0.61) | 1 (0.31) | 0 (0) | 0 (0) | 0.27 |
| Gene Expression | 1,774 (99.05) | 10 (0.56) | 7 (0.39) | 0 (0) | 0 (0) | <0.001 |
| Pharmacodynamics | 1,835 (98.92) | 1 (0.05) | 4 (0.22) | 11 (0.59) | 4 (0.22) | 0.013 |
| Pharmacokinetics | 1,983 (99.20) | 3 (0.15) | 3 (0.15) | 10 (0.50) | 0 (0) | 0.001 |
| Skin (Hyperpigmentation, Dyschromia,  Photosensitivity Disorder, Sun  Exposure, Pigmentation Disorder) | 170 (95.51) | 5 (2.81) | 3 (1.69) | 0 (0) | 0 (0) | 0.011 |
| Smoking (Nicotine Dependence,  Nicotine Patch, Nicotine  Replacement, Nicotine Withdrawal,  OPRM1, Smoking Cessation) | 1,079 (97.21) | 4 (0.36) | 12 (1.08) | 7 (0.63) | 8 (0.72) | <0.001 |
| Vitamin D (+Deficiency, +Metabolism,  +Response, +Supplements, Vitamin D3) | 367 (93.86) | 9 (2.30) | 15 (3.84) | 0 (0) | 0 (0) | <0.001 |

Supplemental Table 2. For the random sample only, characteristics of trials by presence of racial inclusion type. The p-value compares those trials with no exclusions with those trials with exclusions. Due to small sample sizes in some cells, it was not necessarily meaningful to compare the trials with different types of exclusions to each other. We excluded missing data from calculations of p-values.

|  | **Open to more than 1 race**  **n (row %)** | **White**  **Only**  **n (row %)** | **Black**  **Only**  **n (row %)** | **Asian**  **Only**  **n (row %)** | **Hispanic**  **Only**  **n (row %)** | **P-value** |
| --- | --- | --- | --- | --- | --- | --- |
| **Number** | 10,202 | 14 | 48 | 20 | 30 |  |
| **Year Opened** |  |  |  |  |  | 0.71 |
| Before 1995 | 110 (100) | 0 (0) | 0 (0) | 0 (0) | 0 (0) |  |
| 1995-1999 | 464 (99.15) | 1 (0.21) | 2 (0.43) | 0 (0) | 1 (0.21) |  |
| 2000-2004 | 1,794 (99.12) | 1 (0.06) | 10 (0.55) | 1 (0.06) | 4 (0.22) |  |
| 2005-2009 | 4,477 (98.87) | 5 (0.11) | 22 (0.49) | 8 (0.18) | 16 (0.35) |  |
| 2010 and later | 3,064 (98.74) | 7 (0.23) | 13 (0.42) | 10 (0.32) | 9 (0.29) |  |
| Missing | 293 (99.32) | 0 (0) | 1 (0.34) | 1 (0.34) | 0 (0) |  |
| **Funding Agency** |  |  |  |  |  | 0.026 |
| Industry | 2,997 (99.37) | 4 (0.13) | 2 (0.07) | 13 (0.43) | 0 (0) |  |
| NIH | 1,088 (98.64) | 2 (0.18) | 7 (0.63) | 1 (0.09) | 5 (0.45) |  |
| U.S. Federal Government | 232 (99.15) | 0 (0) | 1 (0.43) | 0 (0) | 1 (0.43) |  |
| Combination of the above | 2,937 (98.89) | 4 (0.13) | 17 (0.57) | 3 (0.10) | 9 (0.30) |  |
| Other | 2,948 (98.56) | 4 (0.13) | 21 (0.70) | 3 (0.10) | 15 (0.50) |  |
| **Study Type** |  |  |  |  |  | 0.53 |
| Expanded Access | 13 (100) | 0 (0) | 0 (0) | 0 (0) | 0 (0) |  |
| Interventional | 8,409 (98.87) | 13 (0.15) | 37 (0.44) | 17 (0.20) | 29 (0.34) |  |
| Observational | 1,780 (99.11) | 1 (0.06) | 11 (0.61) | 3 (0.17) | 1 (0.06) |  |
| **Intervention** |  |  |  |  |  | <0.001 |
| Behavioral | 914 (94.72) | 2 (0.21) | 24 (2.49) | 5 (0.52) | 20 (2.07) |  |
| Biological | 737 (100) | 0 (0) | 0 (0) | 0 (0) | 0 (0) |  |
| Device | 677 (100) | 0 (0) | 0 (0) | 0 (0) | 0 (0) |  |
| Dietary Supplement | 176 (98.88) | 1 (0.56) | 1 (0.56) | 0 (0) | 0 (0) |  |
| Drug | 5,191 (99.41) | 9 (0.17) | 7 (0.13) | 12 (0.23) | 3 (0.06) |  |
| Genetic | 50 (96.15) | 1 (1.92) | 0 (0) | 1 (1.92) | 0 (0) |  |
| Other | 1,813 (98.69) | 1 (0.05) | 14 (0.76) | 2 (0.11) | 7 (0.38) |  |
| Procedure | 564 (99.65) | 0 (0) | 2 (0.35) | 0 (0) | 0 (0) |  |
| Radiation | 80 (100) | 0 (0) | 0 (0) | 0 (0) | 0 (0) |  |
| **Phase** |  |  |  |  |  | <0.001 |
| Phase 0 | 72 (98.63) | 0 (0) | 1 (1.37) | 0 (0) | 0 (0) |  |
| Phase 1 | 1,430 (98.76) | 3 (0.21) | 6 (0.41) | 9 (0.62) | 0 (0) |  |
| Phase 1 \| Phase 2 | 498 (98.81) | 1 (0.20) | 2 (0.40) | 2 (0.40) | 1 (0.20) |  |
| Phase 2 | 2,331 (99.79) | 1 (0.04) | 1 (0.04) | 1 (0.04) | 2 (0.09) |  |
| Phase 2 \| Phase 3 | 165 (99.4) | 0 (0) | 0 (0) | 0 (0) | 1 (0.6) |  |
| Phase 3 | 1,282 (99.46) | 0 (0) | 5 (0.39) | 2 (0.16) | 0 (0) |  |
| Phase 4 | 754 (98.82) | 2 (0.26) | 5 (0.66) | 0 (0) | 2 (0.26) |  |
| Other | 3,670 (98.26) | 7 (0.19) | 28 (0.75) | 6 (0.16) | 24 (0.64) |  |
| **Age groups** |  |  |  |  |  | 0.20 |
| Adults or Seniors | 7,864 (98.83) | 14 (0.18) | 35 (0.44) | 18 (0.23) | 26 (0.33) |  |
| Children only | 1,735 (99.31) | 0 (0) | 10 (0.57) | 1 (0.06) | 1 (0.06) |  |
| All ages | 603 (98.85) | 0 (0) | 3 (0.49) | 1 (0.16) | 3 (0.49) |  |
| **Gender** |  |  |  |  |  | <0.001 |
| Both | 8,839 (99.20) | 8 (0.09) | 30 (0.34) | 16 (0.18) | 17 (0.19) |  |
| Female | 962 (97.17) | 5 (0.51) | 11 (1.11) | 0 (0) | 12 (1.21) |  |
| Male | 401 (96.86) | 1 (0.24) | 7 (1.69) | 4 (0.97) | 1 (0.24) |  |
| **Region Opened** |  |  |  |  |  | <0.001 |
| Midwest | 1,435 (98.97) | 3 (0.21) | 9 (0.62) | 2 (0.14) | 1 (0.07) |  |
| Northeast | 1,858 (98.83) | 3 (0.16) | 10 (0.53) | 2 (0.11) | 7 (0.37) |  |
| South | 2,513 (98.43) | 3 (0.12) | 23 (0.90) | 1 (0.04) | 13 (0.51) |  |
| West | 1,319 (98.07) | 1 (0.07) | 4 (0.30) | 12 (0.89) | 9 (0.67) |  |
| Multi-region | 2,912 (99.79) | 2 (0.07) | 2 (0.07) | 2 (0.07) | 0 (0) |  |
| Missing | 165 (98.21) | 2 (1.19) | 0 (0) | 1 (0.60) | 0 (0) |  |

Supplemental Table 3. For the random sample only, demographic characteristics of the ZIP-codes by racial inclusion type. Trials with missing ZIP-code data were excluded. SD=standard deviation, IQR=inter-quartile range, n=Number of trials with non-missing data. The statistics summarize the percents among ZIP-codes.

|  | **Open to more than 1 race** | **White Only** | **Black Only** | **Asian Only** | **Hispanic Only** | **Kruskal-Wallis**  **P-value** |
| --- | --- | --- | --- | --- | --- | --- |
| Percent responding that they are White alone in ZIP-code | n=7,846 | n=12 | n=38 | n=11 | n=22 |  |
| Mean (SD) | 57.1% (20.3%) | 58.6% (20.7%) | 43.9% (25.5%) | 54.1% (21.2%) | 39.1% (27.0%) | 0.0003 |
| Median (IQR) | 59.6% (45.5%,71.2%) | 65.7% (38.2%,72.4%) | 42.4% (30%,64.7%) | 59.9% (42%,62.7%) | 33.8% (18%,64.3%) |  |
| Percent responding that they are Black/African American alone in ZIP-code | n=7,846 | n=12 | n=38 | n=11 | n=22 |  |
| Mean (SD) | 17.1% (17.6%) | 20.3% (18.1%) | 33.5% (25.9%) | 6.4% (5.8%) | 10% (8.1%) | 0.0001 |
| Median (IQR) | 11.5% (5%,22.1%) | 14.8% (5.8%,32%) | 31.1% (14.8%,42.6%) | 5.2% (1.5%,9.4%) | 7.6%  (3.3%,14.8%) |  |
| Percent responding that they are Asian alone in ZIP-code | n=7,846 | n=12 | n=38 | n=11 | n=22 |  |
| Mean (SD) | 9.7% (9.4%) | 10.6% (4.9%) | 7.2% (6.1%) | 21.8% (15.6%) | 6.2% (6.3%) | 0.0001 |
| Median (IQR) | 7.1% (3.9%,12.4%) | 11.2% (7.5%,14.4%) | 4.3% (2.2%,14%) | 17.2% (14.8%,36.4%) | 3.8%  (2.1%,6.7%) |  |
| Percent responding that they are Hispanic alone in ZIP-code | n=7,846 | n=12 | n=38 | n=11 | n=22 |  |
| Mean (SD) | 13.2% (14.1%) | 7.6% (11.6%) | 13% (17%) | 11.4% (7.9%) | 42.3% (28.9%) | 0.0001 |
| Median (IQR) | 9.1% (4.6%,15.5%) | 3.3% (2.7%,6.9%) | 6.1% (2.7%,14.2%) | 8.1% (6%,14.6%) | 48.4% (15.7%,69.6%) |  |
| Percent responding that they speak English only in ZIP-code | n=7,796 | n=12 | n=38 | n=11 | n=22 |  |
| Mean (SD) | 76.3% (13.8%) | 78.9% (12.5%) | 77.7% (17.4%) | 61% (20.1%) | 53.6% (23.9%) | 0.0001 |
| Median (IQR) | 78.3% (70.2%,85.9%) | 83.2% (76.5%,85.3%) | 81% (73.2%,91.9%) | 64.4% (44.9%,72.9%) | 56.4% (36.2%,73.6%) |  |
| Percent responding that they speak English less than “very well” in ZIP-code | n=7,796 | n=12 | n=38 | n=11 | n=22 |  |
| Mean (SD) | 8.4% (7.5%) | 6.3% (6.4%) | 9.2% (11.2%) | 17.8% (11.9%) | 19.8% (14%) | 0.0001 |
| Median (IQR) | 6.5% (3.5%,10.9%) | 4.5% (2.4%,6.4%) | 4.7% (2.3%,9.7%) | 12.6% (7.3%,32.1%) | 19.7%  (6.6%,28%) |  |
| Poverty Rate in ZIP-code | n=7,666 | n=12 | n=36 | n=11 | n=22 |  |
| Mean (SD) | 22% (15.2%) | 29.3% (18.5%) | 25.1% (18.6%) | 16.7% (7.3%) | 20.7% (11.5%) | 0.27 |
| Median (IQR) | 18.2% (11.6%,29.3%) | 31.3% (16.5%,39.2%) | 22.3% (13.9%,31.2%) | 13.8% (11.1%,21.9%) | 20%  (7.8%,30.3%) |  |

Supplemental Table 4: Multiple logistic regression of having a single race/ethnicity requirement (n=18,488 of 19,199 included). We excluded those opened before 1994 (151) or with missing year (n=461). We excluded expanded access (n=7) and radiation (n=92) trials since they had no single race/ethnicity inclusion criteria.

| Variable | Odds Ratio | 95% confidence Interval | P-value |
| --- | --- | --- | --- |
| Year | 1.04 | (1.01, 1.08) | 0.024 |
| Funding Agency |  |  |  |
| Industry | Reference |  |  |
| NIH | 0.84 | (0.50, 1.43) | 0.531 |
| US Federal Government | 0.30 | (0.10, 0.86) | 0.025 |
| Combination of above | 0.67 | (0.44, 1.01) | 0.058 |
| Other | 0.60 | (0.39, 0.91) | 0.016 |
| Study type |  |  |  |
| Expanded Access | NA |  |  |
| Interventional | Reference |  |  |
| Observational | 0.92 | (0.61, 1.39) | 0.71 |
| Intervention Type |  |  |  |
| Behavioral | Reference |  |  |
| Biological | 0.02 | (<0.01, 0.16) | <0.001 |
| Device | 0.06 | (0.02, 0.19) | <0.001 |
| Dietary Supplement | 0.34 | (0.22, 0.53) | <0.001 |
| Drug | 0.17 | (0.12, 0.25) | <0.001 |
| Genetic | 0.27 | (0.06, 1.11) | 0.069 |
| Other | 0.28 | (0.19, 0.43) | <0.001 |
| Procedure | 0.16 | (0.08, 0.36) | <0.001 |
| Radiation | NA |  |  |
| Study Phase |  |  |  |
| Phase 0 | Reference |  |  |
| Phase1 | 1.01 | (0.35, 2.94) | 0.98 |
| Phase 1/Phase 2 | 0.52 | (0.15, 1.80) | 0.31 |
| Phase2 | 0.38 | (0.13, 1.15) | 0.088 |
| Phase 2/Phase 3 | 1.04 | (0.29, 3.70) | 0.95 |
| Phase 3 | 0.90 | (0.30, 2.73) | 0.86 |
| Phase 4 | 1.96 | (0.66, 5.82) | 0.22 |
| Other | 1.14 | (0.41, 3.17) | 0.80 |
| Age group |  |  |  |
| Adults or Seniors | Reference |  |  |
| All ages | 0.82 | (0.57, 1.16) | 0.26 |
| Children only | 1.29 | (0.85, 1.97) | 0.23 |
| Eligible gender |  |  |  |
| Both | Reference |  |  |
| Female | 2.53 | (1.92, 3.33) | <0.001 |
| Male | 3.07 | (2.07, 4.54) | <0.001 |
| Region of U.S. |  |  |  |
| Midwest | Reference |  |  |
| Northeast | 0.88 | (0.60, 1.27) | 0.49 |
| South | 1.34 | (0.95, 1.88) | 0.099 |
| West | 1.45 | (1.00, 2.10) | 0.049 |
| Multiple regions | 0.43 | (0.26, 0.73) | 0.002 |
| Missing region | 1.68 | (0.80, 3.52) | 0.17 |

NA= Not applicable. Radiation and expanded access trials omitted from the regression since no such trials had exclusions.

**Supplemental Figure 1. Trial identification diagram.**

Trials identified through

random search
(n = 10,361)

Trials identified through

enriched targeted search
(n = 10,095)

Trials after duplicates removed
(n = 19,246)

Trials examined
(n = 19,199)

Records excluded due to missing eligibility criteria
(n = 47)
